# A measure of knowledge flow between specific fields: Implications of interdisciplinarity for impact and funding

**DOI:** 10.1371/journal.pone.0185583

**Published:** 2017-10-09

**Authors:** Seokbeom Kwon, Gregg E. A. Solomon, Jan Youtie, Alan L. Porter

**Affiliations:** 1 School of Public Policy, Georgia Institute of Technology, Atlanta, Georgia, United States of America; 2 Directorate for Education and Human Resources, National Science Foundation, Arlington, Virginia, United States of America; 3 Enterprise Innovation Institute, Georgia Institute of Technology, Atlanta, Georgia, United States of America; 4 Search Technology Inc., Atlanta, Georgia, United States of America; GERMANY

## Abstract

Encouraging knowledge flow between mutually relevant disciplines is a worthy aim of research policy makers. Yet, it is less clear what types of research promote cross-disciplinary knowledge flow and whether such research generates particularly influential knowledge. Empirical questions remain as to how to identify knowledge-flow mediating research and how to provide support for this research. This study contributes to addressing these gaps by proposing a new way to identify knowledge-flow mediating research at the individual research article level, instead of at more aggregated levels. We identify journal articles that link two mutually relevant disciplines in three ways—aggregating, bridging, and diffusing. We then examine the likelihood that these papers receive subsequent citations or have funding acknowledgments. Our case study of cognitive science and educational research knowledge flow suggests that articles that aggregate knowledge from multiple disciplines are cited significantly more often than are those whose references are drawn primarily from a single discipline. Interestingly, the articles that meet the criteria for being considered knowledge-flow mediators are less likely to reflect funding, based on reported acknowledgements, than were those that did not meet these criteria. Based on these findings, we draw implications for research policymakers.

## Introduction

Promoting interdisciplinary knowledge flow has been a stated aim of many research programs and scientific communities over the past several decades. Studies, however, show that interdisciplinary research is less likely to receive research funding than is discipline-oriented research. Porter and Rossini [1] found interdisciplinary research proposals less likely to be favored in peer-review processes, which they attributed to the lack of established peer groups. Similarly, Metzger and Zare [2] claimed that the funding decisions of federal research agencies are highly biased toward disciplinary research, even though science policy should arguably be supportive of interdisciplinary research. A recent study by Bromham and Dinnage [3] shows that the funding success of interdisciplinary research proposals in Australia was lower than that for disciplinary research proposals.

Surprisingly, it has been empirically less clear whether interdisciplinary research conveys greater influence on subsequent research. It is especially unclear if one is interested in establishing a research agenda that uses targeted institutional support to promote knowledge flows between particular fields rather than interdisciplinary writ large (i.e., without specifying the particular fields). Finding answers to such questions is not trivial, because capturing interdisciplinary knowledge exchange between specific fields is itself not trivial.

Existing approaches to measuring interdisciplinary knowledge flow look broadly across all disciplines. Indices of interdisciplinarity, such as diversity metrics, integration, diffusion, and specialization scores [4–9], offer ways to quantify relatedness among different scientific disciplines using bibliometric information pertaining to selected compilations of academic publications [10, 11], as well as patents [12]. Science mapping visualizations help position scientific fields vis-à-vis other fields and show where research contributions fit [13].

A limitation of these approaches is that they do not readily enable focus on the knowledge interchange patterns between particular fields. If one has questions about the extent to which knowledge is flowing between two particular disciplines, approaches that indicate interdisciplinarity based on citation diversity across all disciplines may even mislead. One can imagine a paper receiving a high score for interdisciplinarity (i.e., integration score) because it draws on a broad range of fields, but not drawing much at all on the disciplines of interest.

In the present paper, we explore novel ways to indicate the degree of mediating interdisciplinary knowledge exchange between specific disciplines by assessing individual research journal articles. In order to show how the proposed methodology can be useful in examining the interdisciplinary knowledge exchange between specific fields, we ask the following questions: (1) Does interdisciplinary research generate more impactful knowledge? (2) How likely is such interdisciplinary research to have been nourished through research funding?

As a case study in the use of these new indicators of cross-disciplinary knowledge exchange, we look at knowledge flow between two disciplines: Cognitive Science (**CogSci**) and Educational Research (**ED**). The measures we propose in this paper could be applied to any pair of disciplines (and extended to more than two). CogSci and ED offer a special case to study questions about knowledge interchange, for they share substantially overlapping research topics and agendas, utilizing for the most part the methodologies of the social and behavioral sciences. Surprisingly, these two fields have had relatively limited systematic research intersections. Cognitive Scientists and Educational Researchers generally reside in different departments, in different schools, within a university, receive different degrees, belong to different professional societies, and, pointedly submit to different journals. Youtie and colleagues [14] found that only about 1 or 2 percent of papers appearing in Cognitive Science journals between 1994 and 2014 ever cited any papers that had appeared in Educational research journals, and that between 17 and 26% of Educational Research papers during those years ever cited an article in a Cognitive Science journal, suggesting a surprising lack of systematic contact between two so seemingly similar fields, Indeed, in the past two decades, a series of programs, such as Research on Learning and Education (ROLE) created by the U.S. National Science Foundation (NSF), the Cognition and Student Learning (CASL) program of the U.S. Department of Education, and the Cognitive Studies for Educational Practice (CSEP) program of the James S. McDonnell Foundation were created with a specific aim of promoting research interaction between these two fields. Policy interest in promoting mutual awareness and interchange of knowledge between these fields heavily engaged with learning provides a strong incentive to study how their research literatures connect.

The compilation of extensive data on those research literatures provides us with a venue to devise metrics of knowledge bridging, and, thence, to address the research questions concerning their influence and support. In this paper, we demonstrate how having metrics of knowledge flow between specific disciplines allows us to derive insights about, in this case, connections between Cognitive Science and Educational research, that we could not obtain were we to use broader measures of interdisciplinarity. First, we identify the journal articles that mediate the knowledge flow between two target disciplines using the new method. Then, we seek to assess the extent to which such articles exert influence, as measured by accruing citations. Finally, we examine articles gathered from the Web of Science (WoS) to compare their funding acknowledgements to comparison articles—to investigate to what degree the research that has led to such knowledge-mediating research had received support.

## Three types of interdisciplinary knowledge-flow mediators

**Fig 1:**
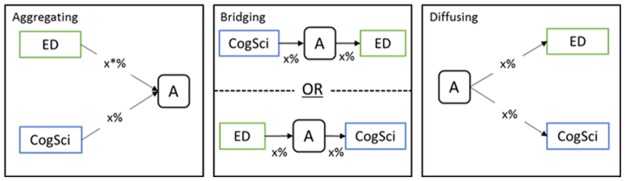
Definition of three types of KMEDs. Note: Aggregating-Type (A-type): More than x% of references of the article A are ED related sources and another more than x% of the references of the article A are CogSci related. Bridging-Type (B-type): More than x% of references comes from CogSci (ED) Sources while more than x% of the publications that cited the article A are published in ED (CogSci)-related sources. Diffusing-Type (D-type): More than x% of publications that cited the article A were published in CogSci related sources and another more than x% of publications citing the article A were published in ED related sources.

We identify three ways by which articles can serve as knowledge-flow mediators (hereafter, **KMED**) between fields, defined at the granular, article level and based on citation information: Aggregating (hereafter, **A-type**) articles draw upon knowledge from multiple fields of interest; Diffusing (hereafter **D-type**) articles are those that have proven influential in multiple fields; and Bridging (hereafter **B-type**) articles draw on knowledge from one particular field and influence another. These are not mutually exclusive. We operationalize each KMED type in terms of disciplinary categories of the cited references and publications that cite the article of interest. Citation analysis allows us to measure such knowledge flow between specific fields in fairly specific ways, though, to be sure, we fully recognize the limitations and dangers in using citation as a proxy for influence. Fig 1 illustrates the definitions of the three types of KMEDs.

We define a target article as A-type if a given percentage, x, of its cited references are to publications from one particular discipline of theoretical interest while another x% of its references are to publications of another discipline of interest. For example, when we set x = 10% and the disciplines of interest are CogSci and ED, we categorize an article as A-type if at least 10% or more of its references are to articles appearing in CogSci sources (i.e., journals or conference proceedings) and at least another 10% are to those appearing in ED sources. We classify a target article as D-type if x% or more of its received citations, that is, the articles that cite the target article, are from articles in sources in one of the disciplines of interest, and x% from articles in another discipline of interest. This can be likened to a reverse of the aggregation of knowledge—influencing rather than being influenced by the knowledge of multiple fields. Finally, we categorize a target article as B-type if x% or more of that article’s own *cited references* are to sources that are related to one discipline and x% of its *received citations* are from sources that are related to another discipline of interest. For example, if at least 10% of a journal article’s own references are to articles published in CogSci journals, and at least 10% of the articles that subsequently cite the target article appear in ED journals, then the target article would be consider a B-type KMED, for it would have bridged the fields of CogSci and ED. We recognize that our evidence of an article that cites publications of one field, being itself cited by publications in the other field, is only suggestive of knowledge transfer potential, not definitive. However, papers that draw overwhelmingly upon sources related to a single field, and are cited almost solely by the publications in the same field, are unlikely to be contributing to knowledge transfer to that other field. By the definition, the three types of KMED are not mutually exclusive; an article can be categorized into multiple types of KMED.

A study by Porter and Rafols [6] posits interdisciplinary research as research that integrates knowledge from different disciplines to solve complex problems. KMEDs operationalize this definition of interdisciplinarity in a more targeted manner than those used in prior studies [15, 16] in that the KMED measures are constructed for interdisciplinary knowledge exchange between specific fields. First, the A-type measure captures the nature of research outcomes that were originated from integration of knowledge in two different disciplines (in this case, CogSci and ED). Hence, the operational definition of the A-type KMED measure is coherent with the definition of interdisciplinarity as knowledge integration. Second, B- and D-type measures help one to capture the characteristics of research that potentially contributes to promoting interdisciplinary knowledge integration in CogSci and ED. B-type examines the extent to which the knowledge of one field is connected to another through the research outcome of interest. Meanwhile, D-type is designed to indicate whether the research outcome of interest can promote integration of knowledge in the two fields of interest by becoming a common knowledge input for future research in the two fields.

Determining the value of “x” in finding the KMED articles poses both theoretical and empirical choices. In an earlier investigation of the disciplinary attributes of the journal *Cognitive Science*, Schunn and Crowley [17] considered a field to have had a minor influence on the writing of an article if 5% of its references were to articles appearing in journals from that field, and a major influence if a higher threshold were from such journals. Following in the spirit of that paper, we set x at 10% as an intermediate, exploratory criterion. In the Appendix, we report findings with x set at 0 (x>0%), 5, and 20% to check sensitivity of our findings to the variations in x. The sensitivity test shows findings consistent with that obtained with x = 10%, though, as expected, the number of articles meeting the criterion drops as x is increased.

In the next section, we apply the KMED measures to investigate whether the KMED articles generate more impactful knowledge on subsequent research and whether they are likely to have resulted from research funding, for the case of CogSci and ED. We also take this opportunity to investigate the role played by what Youtie and her colleagues [14] refer to as “Border fields,” disciplines existing between CogSci and ED. These fields include educational psychology, the learning sciences, human development, learning technology and human-computer interaction, and applied linguistics. Youtie and her colleagues suggested that, though they are knowledge producing fields in their own rights, these Border fields may also play a role in mediating or bridging the flow of knowledge between CogSci and ED.

## Methods

### Data

We start with searches for articles that were published in 1994, 1999, 2004, 2009, and 2014 in journals that are dedicated to CogSci, ED, and Border fields. We chose to use these as our sample cohorts because the increments span the year 2000, a watershed moment, as noted above, in efforts to foster connections between CogSci, ED, and Border fields [14]. We draw data from the Web of Science (**WoS**) Core Collection Citation Indexes. Web of Science Categories (**WCs**) provide a starting point for selection of journals covering our interests relating to the exchange of research knowledge among ED, CogSci, and Border fields. These selections result in a set of 177 journals Youtie et al., [14] provide details on the process). These include:

- 66 ED journals, including ones in the WC “Education and Education Research,” supplemented by journals in WCs relating to education in Scientific, Technical, Engineering, and Math (STEM) disciplines.
- 42 CogSci journals, guided by Goldstone and Leydesdorff [18] analysis identifying highly cited journals in the flagship journal, *Cognitive Science*. We augmented with CogSci journals introduced since 2006, de-emphasizing those with clinical or biological priorities. All appear in WCs relevant to CogSci—Experimental Psychology, Linguistics, Artificial Intelligence, and Neuroscience (very selectively).
- 69 Border field journals, including 39 from Educational Psychology, 23 from Learning Technology/Human Computer Interaction, and 7 from Applied Linguistics WCs.

We limit our sample to WoS document types “conference proceeding/journal articles” or journal articles, while excluding review articles because we focus on papers that contain novel research elements. Our search yields 32,121 articles. Then, we categorize the cited references and the received citations of each article into CogSci, ED, Border, and Other. Since WoS does not provide category information for the cited/citing references, we employ a multi-stage categorization process that combines an automatic journal-title pattern matching algorithm and manual classification. We first categorize the cited/citing publications’ sources that are in the 177-journal list. Second, we apply a thesaurus that associates other journals indexed by WoS (and some non-journal sources) to the four categories for this study (CogSci, ED, Border, and Other). These first two stages categorize about half of the total 1.4 million cited references. Third, we conduct a human-aided categorization for the sources not yet categorized. The uncategorized cited/citing articles’ sources are processed by an automatic categorization program that recognizes the specific pattern in the title of the citing/cited publications’ source. For instance, an article that is published in the journal of Educational Psychology is categorized into the Border field because the name of the journal has both an ED -elated word (i.e. Educational) and a CogSci-related word (“Psychology”). The categorization results are randomly sampled and reviewed by experts. The categorization routine is modified, according to expert judgments, until the categorization outcome exceeds 90% agreement with the expert’s assignment. These combinatorial categorization procedures, all together, classify about 85% of the cited references and 96% of the articles that cite the 32,121 articles in the sample. The dataset contains 15,455 CogSci (48%), 9,082 ED (28%), and 7,584 (24%) Border field articles. We, then, classify the collected articles into the three types of KMEDs or non-KMEDs.

## Results

### Prevalence of KMED articles

Table 1 summarizes the number and percentage of articles that can be categorized as KMED, by KMED type, discipline, and year of publication. Even at this broad level of analysis, the measure affords us insights. First, collapsing across fields, we see that only a minority—about 25%–of the 32,121 articles met the criterion for fitting at least one KMED type. That most research papers are relatively disciplinary should not be surprising, given the disciplinary orientation of the great majority of journals. Moreover, it supports the notion that these literatures are surprisingly separate, despite their shared “learning” questions.

**Table 1:** Number of articles in CogSci, ED, and border journals, by KMED type and year.

CogSci	Non-KMED		KMED		A-Type		B-Type		D-Type	
Year	Obs	Percent	Obs	Percent	Obs	Percent	Obs	Percent	Obs	Percent
1994	1,740	89	213	11	146	7	199	10	110	6
1999	2,209	91	210	9	131	5	204	8	126	5
2004	2,492	92	226	8	125	5	221	8	162	6
2009	3,720	93	284	7	124	3	276	7	206	5
2014	4,170	96	191	4	165	4	73	2	26	1
Total	14,331	93	1,124	7	691	4	973	6	630	4
ED	Non-KMED		KMED		A-Type		B-Type		D-Type	
Year	Obs	Percent	Obs	Percent	Obs	Percent	Obs	Percent	Obs	Percent
1994	699	77	208	23	152	17	175	19	92	10
1999	1,010	80	258	20	184	15	243	19	125	10
2004	1,178	77	356	23	264	17	326	21	154	10
2009	1,772	77	529	23	398	17	489	21	221	10
2014	2,501	81	571	19	554	18	103	3	15	0
Total	7,160	79	1,922	21	1,552	17	1,336	15	607	7
Border	Non-KMED		KMED		A-Type		B-Type		D-Type	
Year	Obs	Percent	Obs	Percent	Obs	Percent	Obs	Percent	Obs	Percent
1994	370	45	449	55	362	44	335	41	179	22
1999	309	36	549	64	406	47	492	57	282	33
2004	413	40	620	60	479	46	550	53	326	32
2009	964	43	1,257	57	955	43	1,087	49	532	24
2014	1,338	50	1,315	50	1,283	48	168	6	20	1
Total	3,394	45	4,190	55	3,485	46	2,632	35	1,339	18

Second, these percentages varied by field: 56% of Border field articles were KMED, whereas 20% of ED articles and only 7% of CogSci articles were. A 3x5 Analysis of Variance (ANOVA), with the 3 levels of field (ED, CogSci, and Border) crossed with 5 levels of year (1994, 1999, 2004, 2009, and 2014) showed there to be a significant main effect of field (F(2, 32106) = 3695, p < .001, η^2^ = 0.2). Indeed, when we simply look at the raw numbers of KMED articles, we can see that the number of KMED articles appearing in Border field journals that link CogSci and ED is greater than the number of such articles appearing in Ed and CogSci journals combined. This underscores the special role played by Border fields, as discussed in Youtie et al.,[14]. Finally, we further see a significant main effect of year (F(4,32106) = 40, p < .001, η^2^ = 0.005) for the number of KMED articles published, indicating that across these three fields there was a general increase in the number of KMED articles, though this increase was not level across fields.

Focusing on the raw number of A-type KMED articles, Table 1 show that their frequency in CogSci journals went up by 19 articles from 1994 to 2014, whereas during that period there were an additional 400 A-Type articles appearing in ED journals, and an additional 900 in Border Field journals. As can be seen in the table, this increase in frequency of KMED articles reflects the fact that there has been a general increase in the number of publications of all kinds in these fields, as indeed has been observed of science publication across all fields (c.f., 6). Pointedly, the proportion of A-type KMED articles from 1994 to 2014 dropped slightly from 7% to 4% in CogSci and stayed roughly level at about 17 to 18% in ED articles and rose slightly from 44% to 49% in Border Field articles.

Table 1 further shows that the prevalence of the different KMED types differed somewhat between fields. For Border field and ED articles, A-type KMED articles were modal, whereas for CogSci articles, B-type were modal. Such differences notwithstanding, the relative trends were roughly the same for the different KMED types for each field. That is, Border field articles were far more likely to show each KMED pattern than were the CogSci or ED articles, and for all fields, the trend over the years was no increase in the proportion of articles that were KMED. But there were suggestive differences, especially when comparing the CogSci and ED articles. Whereas ED articles were about four or five times more likely to be A-Type KMED, indicating that they were far more likely to draw on both ED and CogSci literatures, they were only about twice as likely to be D-type. As Table 1 shows, more CogSci articles qualified as D-Type than did ED articles. That is, more articles in CogSci journals are cited by both fields than were articles in ED journals.

Finally, we note that about 58% of the KMED articles connecting ED and CogSci were published in Border field journals. These findings again are consistent with the notion that the Border fields mediate knowledge exchange between CogSci and ED.

### Relationship among KMEDs

**Fig 2:**
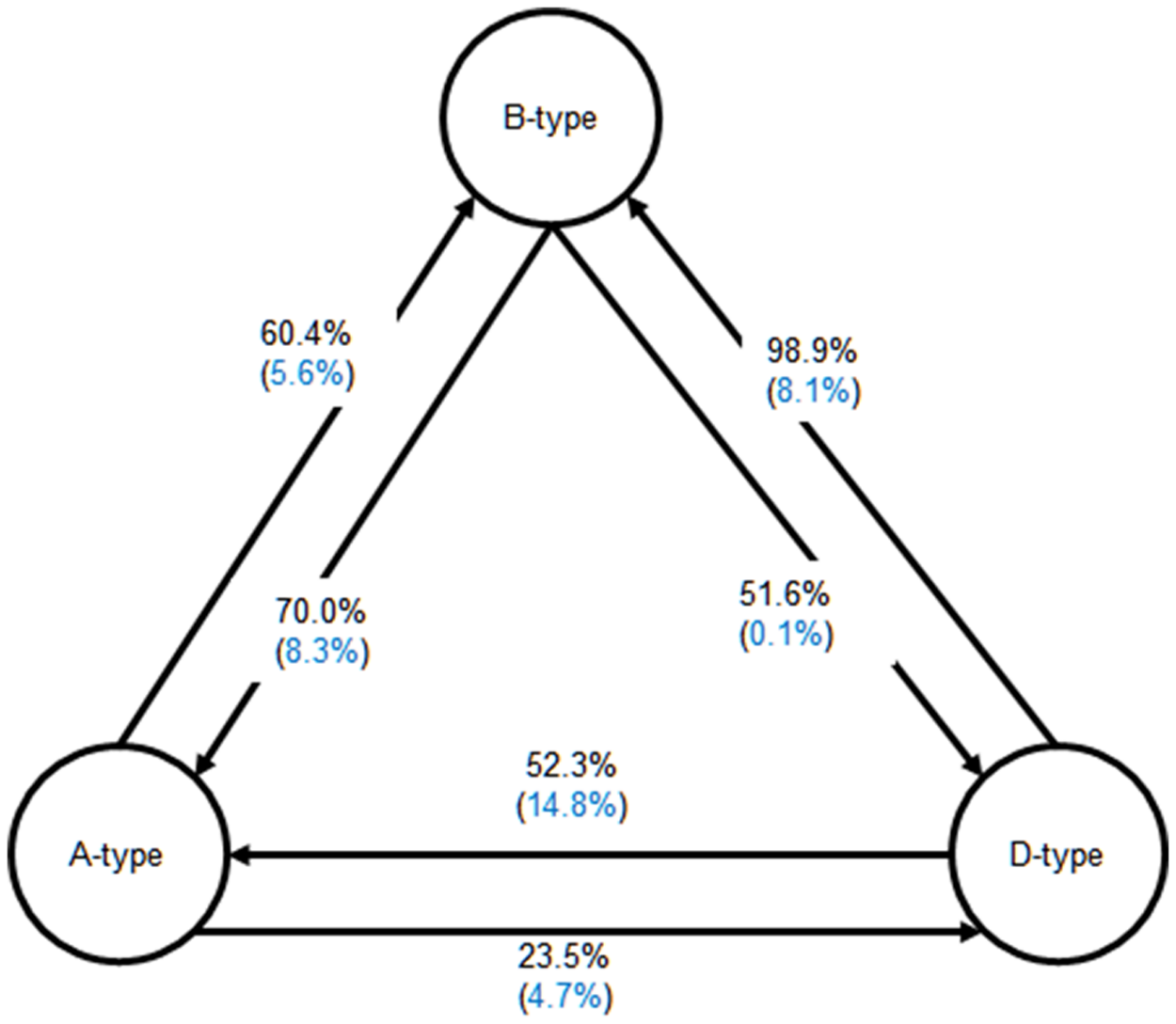
Relationship among the three types of KMEDs. Black: % of the (starting point) KMED articles that are also categorized into the (ending-point) KMED. Blue: % of the articles that are not the (starting point) KMED but that are categorized into the (ending-point) KMED.

There is, of course, some definitional overlap between KMED types—B-type, for example, are codefined to an extent with A-type and D-type. At this point, it is worth asking about the extent to which there is empirical overlap. To what extent to articles that reflect one cross-disciplinary attribute tend to have others too? Are articles that cite across disciplinary bounds in turn cited by articles in those disciplines? And are articles from one field that are heavily cited in another field also more likely to have drawn on both fields in their references? Fig 2 shows the percentage of each type of KMED that is also categorized into the other types of KMEDs. About 60% of the A-type articles also appear as B-type, whereas only 6% of the non-A-type articles (including B-, and D-type but not A-type articles and non-KMED articles) are categorized into B-Type (this difference between non-A-Type and A-type in terms of their likelihood of being categorized as B-type is statistically significant, z = -100, p < .001). About 24% of the A-type KMEDs are classified into D-type, whereas only 5% of the non-A-type articles are the D-type KMED (this difference between non-A-Type and A-type in terms of their likelihood of being categorized as D-type is statistically significant, z = -48, p < .001). It would appear, then, that articles that draw on articles from both fields are more likely to be cited by articles in both fields and more likely to bridge the knowledge exchange between the two fields than are those articles that do not.

We conduct a similar analysis for the B-, and D-type articles. About 70% of the B-type articles are categorized into A-type, while only 8% of the non-B-Type articles are A-type KMEDs (this difference between non-B-Type and B-type in terms of their likelihood of being categorized as A-type is statistically significant, z = -100, p < .001). Also, about 52% of the B-type articles are D-type KMEDs, whereas only 0.1% of the non-B-type articles are D-type KMEDs (this difference between non-B-Type and B-type in terms of their likelihood of being categorized as D-type is statistically significant, z = -120, p < .001). Finally, about 52% of the D-type KMEDs are A-type KMEDs, whereas 15% of the non-D-type KMEDs are A-type KMEDs (this difference between non-D-Type and D-type in terms of their likelihood of being categorized as A-type is statistically significant, z = -48, p < .001). Notably, about 99% of the D-type articles are also B-type KMEDs, whereas 8% of the non-D-type articles are categorized into the B-type (this difference between non-D-Type and D-type in terms of their likelihood of being categorized as B-type is statistically significant, z = -120, p < .001).

This finding indicates that the research outputs that draw upon knowledge in CogSci and ED (A-type) are more likely to bridge the knowledge flow between the two disciplines. Likewise, the B-type research outputs that bridge the knowledge flow between the two disciplines are also highly likely to influence following research in both fields. And, D-type research that exerts influence on both disciplines is likely to have incorporated knowledge from both CogSci and ED.

### Research impact and roles of border fields

In order to address the question of whether KMED articles have a greater impact than do non-KMED articles, we look to the number of citations that an article received as a proxy for impact. The KMED measure allows us to ask this question more specifically: Are articles integrating the ED and CogSci literatures more likely to be impactful? We limit our analysis here to comparing A-type KMEDs to non-KMED articles. Comparing the number of received citations by the B- or D-types with that of non-KMED articles is somewhat confounded because these two types are defined by the categories of the publications citing the article of interest.

A-Type KMEDs received disproportionally more citations than did the non-KMEDs (see Appendix 1 for the comparison of the median /median value by publication year with threshold x varied; we note that the trends discussed here do not vary significantly). A 5x3x2 ANOVA, with the 5 levels of year crossed with the 3 levels of field and 2 levels of KMED, shows a significant main effect for year (F(4, 30583) = 370, p < .001, η^2^ = 0.05). There was also a significant main effect for field, with CogSci articles cited a mean of 30 times, ED cited a mean of 9 times, and Border a mean of 12 times (F(2,30583) = 395, p < .001, η^2^ = 0.03).

When we look at A-type vs. non-KMED articles for each field, A-type CogSci articles received a mean of 49 citations, statistically greater than the mean of 28 citations received by the non-KMED CogSci articles (F(1,15012) = 38, p < .001, η^2^ = 0.003). An average A-type ED article received 13 citations while a non-KMED ED article received only 8 citations, also statistically significant (F(1,8702) = 185, p < .001, η^2^ = 0.02). Similarly, a Border field A-type article received 15 citations, whereas an average non-KMED Border article received 8 citations. This difference is also statistically significant at the 0.001 level (F(1,6869) = 235, p < .001, η^2^ = 0.03). The findings that A-type received more citations than non-KMEDs, across the disciplines and years, suggests that research aggregating knowledge from both CogSci and ED exerted more influence on future researchers on average.

**Fig 3:**
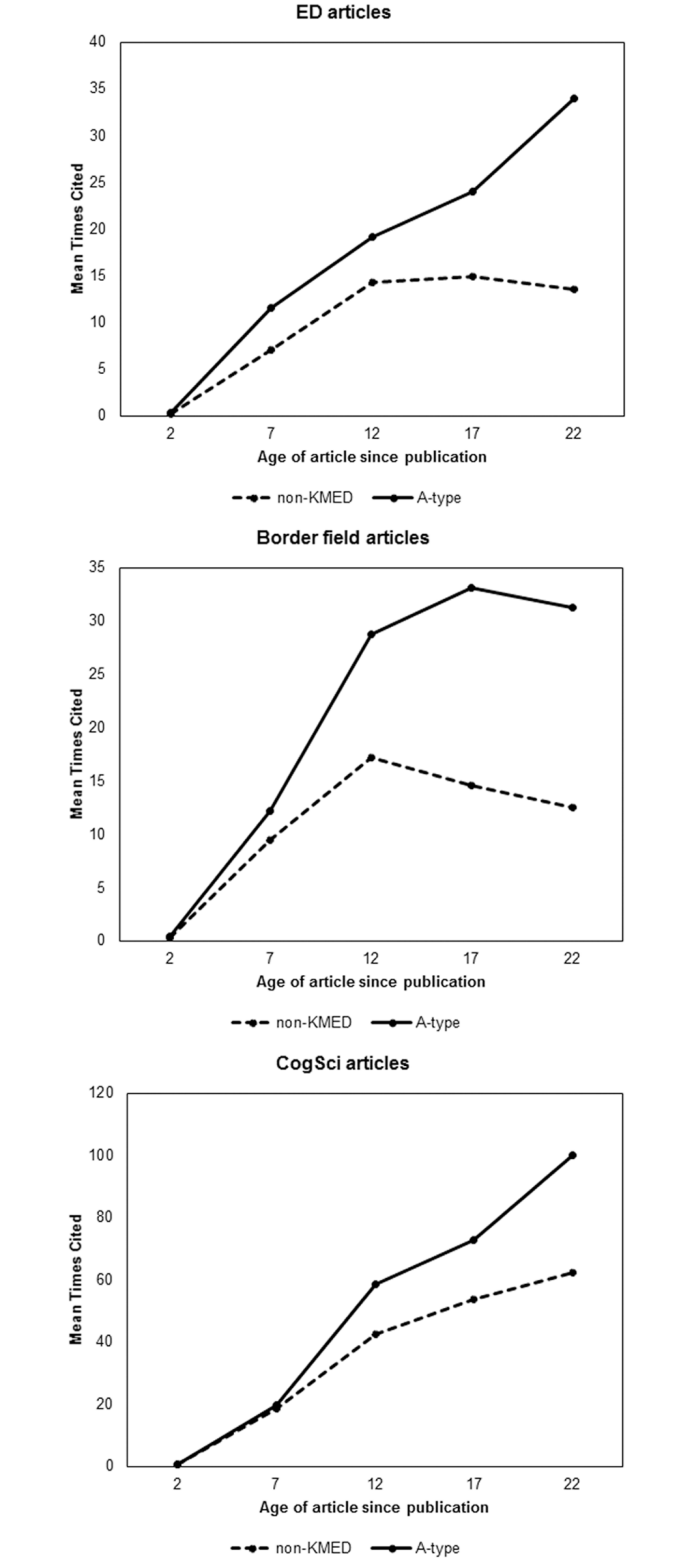
The mean number of citations received by articles published in ED, border, and CogSci journals, by age and KMED status.

Strikingly, Fig 3 show different citation trends of A-type and non-KMED articles by age of article for the three fields (age of article is calculated as 2016-publication year). For articles appearing in Border field journals, those published in 2004 (age = 12), both A-type and non-KMED, accrued roughly twice as many more citations than have those published in 2009 (age = 7), which in turn were cited more often than those published most recently, in 2014 (age = 2). That older articles should accrue more citations, because they have more years available for citing, and then decline as the content becomes obsolete is not surprising. The literature on article citation windows and obsolescence indicates that citation rates tend to flatten after reaching a maximum point, and these differences in citation windows vary by field, with social sciences (including the fields in this paper) having longer citation windows than natural or biological sciences [19–22]. Border field articles appearing in 1994 (age = 22) and 1999 (age = 17) accrued about as many citations as had those published in 2004 (age = 12). We note that whereas Price [22] had found a leveling of citations after about a five-year window for the average field, Fig 3 shows it to have occurred with articles published after 10 years in Border field journals.

Articles appearing in ED journals showed a different pattern. As with the Border Fields articles, the ED articles appearing in 2004 accrued many more citations than had those appearing in 2009, again likely because more years are available for citing in the earlier period. This was true of the 2004 A-type articles (receiving about 50% more citations than had those published in 2009) and the non-KMED articles (receiving twice as many as in 2009). Again, as with the Border field articles, the curve is flat for non-KMED ED articles appearing between 1994 and 2004. By contrast, the articles appearing in ED journals that are A-type show a different pattern. On average, the older articles appear to continue to receive citations: the articles appearing in 1999 received about a third more citations than those appearing in 2004, and those appearing in 1994 received about a third more citation than had those published in 1999. In short, A-type articles are less likely to become obsolete and have longer citation windows.

The citation of CogSci articles diverges even more from de Solla Price’s finding of a general flattening, then declining, of mean citation rates after 5 years. Consistent with the accrual of citations for ED and Border field articles, the non-KMED CogSci articles appearing in 2004 accrued more than twice as many citations as had those appearing in 2009 and the KMED articles received about three times as many. Interestingly, the non-KMED and A-type CogSci articles appearing in 1999 received on average about 25% more citations than had non-KMED and A-type articles appearing in 2004, respectively, and non-KMED articles appearing in 1994 received about 15% more citations than did those appearing in 1999, with A-type articles receiving about a third more citation in 1994 than had those appearing in 1999.

### Funding

In order to examine whether KMED articles were more likely to have arisen from research funding support, we compare the likelihood of having funding acknowledgements in the KMED and non-KMED articles. Because WoS started to record funding acknowledgments from August 2008 (http://wokinfo.com/products_tools/multidisciplinary/webofscience/fundingsearch/ (accessed 11/4/2016)), we limit the dataset for funding analyses to articles published in 2009 and 2014. The coverage of funding acknowledgment information in WoS can be questionable in that it is based on self-reporting by authors and it potentially varies by such factors as research context, researcher’s country, and institutional norms, Nonetheless, Grassano, and Rotolo [23] indicated that WoS data provide reasonable coverage of the funding of research articles by showing that about 93% of U.K funded cancer research’s funding information has been indexed by WoS.

**Fig 4:**
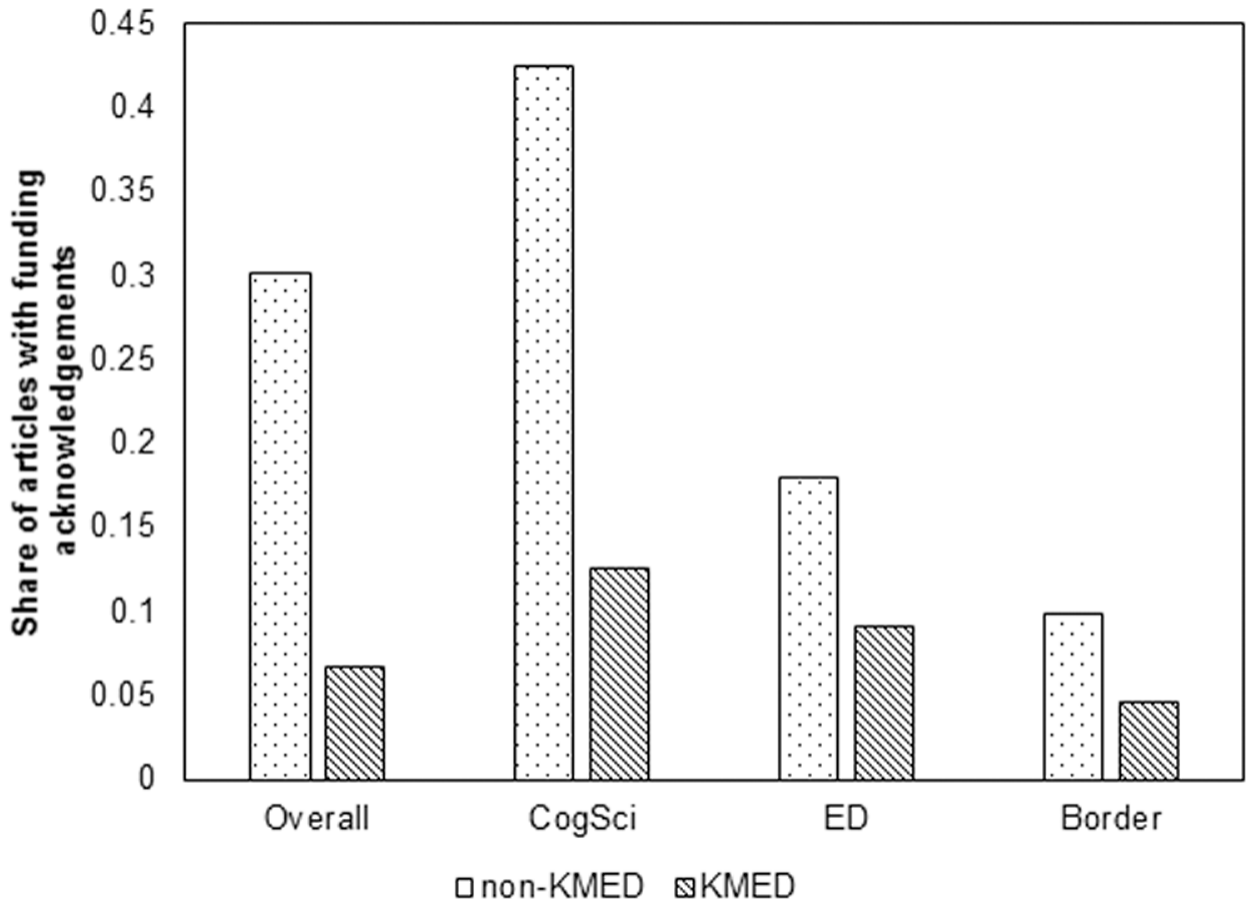
Comparison of share of the articles with funding Acknowledgement by (non-KMEDs vs. KMEDs).

Fig 4 shows that KMED articles were less likely to acknowledge funding than were non-KMED articles on average. About 30% of the non-KMED articles have funding acknowledgements whereas only 7% of the KMED articles do. Interestingly, the Border field articles were significantly less likely to have funding acknowledgements than the CogSci or ED articles for both KMED and non-KMED. [This difference is statistically significant based on the proportion test (for Border vs. CogSci KMEDs, z = 7, p < .001; for Border vs. ED KMEDs, z = 5, p < .001;for Border vs. CogSci Non-KMEDs, z = 29, p < .001; for Border vs. ED non-KMEDS, z = 9, p < .001).]

For non-KMED articles, only about 10% of the Border field articles have funding acknowledgements, whereas 43% and 18% of the CogSci and ED articles do, respectively. For the KMED articles, 5% of those in Border fields have funding acknowledgements, whereas about 13% and 9% of the CogSci and ED articles do. All the observed differences between KMEDs and non-KMEDs with respect to the likelihood of having funding acknowledgements are statistically significant at the 0.001 significance level (proportion test on difference between KMED and non-KMED: Overall (z = -31, p < .001), CogSci (z = -13, p < .001), ED (z = -7, p < .001), Border field (z = -7,p < .001)).

## Discussion and conclusions

In the present study, we develop a new bibliometric method to identify research that mediates cross-disciplinary knowledge flow between specific disciplines. By using the disciplinary categories of the cited references and the categories of citing sources, we provide three indicators that capture different ways of mediating the knowledge exchange between articles across two specific disciplines of interest. A-type represents the nature of research outputs that incorporate knowledge from both fields. B-type represents research outputs that bridge knowledge across the two fields. D-type represents research that influences future research in both fields, as indicated by substantial citation to the articles in question.

We also find that research outputs from Border fields appear to mediate the knowledge exchange between the two disciplines, CogSci and ED. Many of the Border field articles fall into the KMED types. This finding suggests that a discipline that attracts researchers in mutually relevant fields, that have not interacted directly to a high degree, can facilitate discipline level interaction by acting as an intermediary field.

Through the case of CogSci and ED, we show that KMED articles offer influential knowledge. The A-type articles receive significantly more citations from future research than the non-KMEDs do. Our further analysis shows an article that is categorized into one type of KMED is more likely to be classified into other types of KMEDs, which indicates that the three different aspects of knowledge mediation are closely related to each other. Accordingly, we conclude that the research outputs that mediate knowledge flow between two mutually relevant disciplines may exert more influence than those that focus within-field.

The results do not speak to the influence of individual articles, but they do further indicate that KMED articles in ED, CogSci, and Border field journals, on average, have a longer citation window. Findings further suggest that KMED articles appearing in ED and CogSci journals continue to be cited and avoid obsolescence longer. Finally, the results suggest that even non-KMED CogSci articles on average continue to influence later work.

Strikingly, the KMED articles are less likely to report funding acknowledgements than are non-KMEDs. This finding needs to be carefully interpreted as research funding decisions are not made of articles, but of proposals. Because not all the research articles come from research projects that were proposed for research funding in the CogSci and ED case, the difference in the likelihood of containing funding acknowledgement in the article does not conclusively indicate that interdisciplinary research proposals are less likely to be supported than are discipline-oriented ones. We do not know whether the likelihood that a paper is KMED is related to the likelihood that the research proposal from which it was derived was KMED. Moreover, we do not know that the authors of KMED proposals had necessarily been denied funding. It is also plausible that those researchers who conduct research that potentially mediates knowledge exchange between the two fields may not particularly seek research funding. However, considering prior studies [1, 3, 24] that found interdisciplinary research proposals less likely to receive funding than disciplinary research in general, our finding suggests that the more the knowledge-flow mediating characteristics, the less the likelihood of acknowledging research funding. Chubin and Connolly [24] claim that research policymakers need to provide well-crafted funding incentives. Nichols [25] suggests that practicing co-funding for interdisciplinary research through cooperation of program officers can be another way of improving the environment for supporting interdisciplinary research.

Our case analyses in the present study is confined to CogSci and ED research (along with Border field analyses). These fields would seem to be likely to generate B-type articles because of agenda overlaps between the two fields and the potential for some of these journals to orient themselves as being an appropriate place for manuscript submission for broadly positioned papers. At the same time, the prior work of Youtie and colleagues [14] notes that there is little cross-citation between the two fields. Nevertheless, we recommend that this KMED methodology be applied to other fields, particularly those with less presumed overlap or with more distinct research foci. Our research therefore calls for studies of different fields. It is an empirical question whether the results we have found for CogSci and ED would be similar to those found for, say, the interactions of Cellular Biology and Mathematics.

Our study adds to the body of literature on interdisciplinary research. We introduce KMED metrics to distinguish research articles whose citation patterns indicate cross-field knowledge transfer between specific fields of study. Using these measures, we provide empirical results showing that research that mediates knowledge flow between two mutually relevant disciplines has been less likely to receive funding support than non-KMED research, with CogSci and ED as our case. Our findings support the notion that funding decisions implicitly favor research within the “silo” of specific disciplines (here, CogSci and ED, also the Border field that we composed). Our methods and findings also contribute to extending understanding of relations between research impact and interdisciplinarity. According to a study by Larivière and Gingras [15], interdisciplinarity measured by the extent to which an article made citations to a broad range of other disciplines could relate to research impact by the field. Meanwhile, our study shows that the degree to which research that mediates knowledge exchange between “closely related fields” might be positively associated with research impact.

## Limitations and future research

Our research has several caveats that promote opportunities for future research. First, the conclusions are based upon the case of two prominent social/behavioral science fields. Whether they would extend to other fields is an empirical question of some theoretical and practical significance. Future research to apply the knowledge-transfer article classification that we offer to other domains offers promise of pursuing the case for interdisciplinary research.

Second, there are issues in the use of number of received citations from future research for measuring research impact. Various factors affect citation rates, including field publication intensity and norms. In the present case, CogSci articles and citation practices differ considerably from those of ED research. Such field level characteristics have not been taken into account in this study because our comparisons weigh mainly within field between KMED and non-KMED papers.

Third, the samples we used in the present research consist of journal articles that are indexed by WoS. That is, the publications that are not indexed by WoS are systematically excluded from our sample. Hence, the findings of the present research are potentially subject to selection bias. In this case, relatively less ED research than CogSci research would be expected to be captured in a WoS dataset. However, because the WoS captures most of the major journals in many disciplines, we believe that the selection bias is not particularly worrisome for our findings. WoS citation quality is superior and that is essential for our interdisciplinary measurements.

Fourth, for the purpose of present research, we focused only on KMED vs. non-KMED comparisons. However, we believe that studying how the three types of KMEDs exert systematically different research impacts and the likelihood of having funding acknowledgments can extend our understanding of the contribution of research that facilitates knowledge exchange between disciplines. Also, as a study by Yegros-Yegros, Rafols, and D’Este [26] pioneered, examining whether the interdisciplinarity indicated by KMED measures always has a positive relationship with the research impact can be a worthy subject of further study.

Fifth, we used WCs to find journals that are related to CogSci, ED, and Border fields, which could bring noise into the sample in the event that the WoS assigns journals to categories erroneously [27]. However, because we used WCs as a starting point and added manual efforts with experts to select the journals that are dedicated to the three fields, our findings are less likely to suffer seriously from incorrectly assigned WCs.

Last, we believe that the suggested method in the present study can be used in addressing more specific questions. For instance, one can examine the knowledge exchange directions between disciplines of interest by incorporating direction of citation from one discipline to another in the KMED indicators.

## Disclaimers

We note that classification of large numbers of cited and citing papers requires software support (here, using VantagePoint– www.theVantagePoint.com). Considerable data gathering effort is entailed to obtain citing record information (here, for over 700,000 citing records).

## Supporting information

S1 File Search strategy. (DOCX)

S2 File Appendix. (DOCX)
