# Supplementary material for: A measure of knowledge flow between specific fields: Implications of interdisciplinarity for impact and funding
Source: PLoS One. 2017 Oct 9;12(10):e0185583. doi: 10.1371/journal.pone.0185583 (PMC5633153; doi:10.1371/journal.pone.0185583)
Supplement: S1 File — (DOCX) [file pone.0185583.s001.docx]

**Journals assigned to Cognitive Science**

Acta Psychologica

American Journal of Psychology

American Psychologist

Artificial Intelligence

Attention Perception and Psychophysics

Behavioral and Brain Sciences

Biological Cybernetics

Brain and Cognition

British Journal of Psychology

Child Development

Cognition

Cognitive Affective and Behavioral Neuroscience

Cognitive Development

Cognitive Neuropsychology

Cognitive Psychology

Cognitive Science

Current Directions in Psychological Science

Developmental Psychology

Developmental Science

Infant Behavior and Development

Journal of Child Language

Journal of Cognitive Neuroscience

Journal of Experimental Psychology: General

Journal of Experimental Psychology: Human Perception and Performance

Journal of Experimental Psychology: Learning Memory and Cognition

Journal of Memory and Language

Journal of Psycholinguistic Research

Journal of Vision

Language

Language Cognition and Neuroscience

Memory and Cognition

Neural Computation

Neuropsychologia

Perception

Psychological Bulletin

Psychological Review

Psychological Science

Psychometrika

Psychonomic Bulletin and Review

Quarterly Journal of Experimental Psychology

Vision Research

Visual Cognition

**Journals assigned to Education Research**

Academy of Management Learning and Education

American Biology Teacher

American Educational Research Journal

American Journal of Education

American Journal of Physics

Assessment and Evaluation in Higher Education

Biochemistry and Molecular Biology Education

British Educational Research Journal

CBE-Life Sciences Education

Chemistry Education Research and Practice

Comparative Education Review

Computer Applications in Engineering Education

Critical Studies in Education

Early Childhood Research Quarterly

Economics of Education Review

Education Finance and Policy

Educational Evaluation and Policy Analysis

Educational Research Review

Educational Researcher

Educational Studies in Mathematics

Egitim ve Bilim-Education and Science

Elementary School Journal

Environmental Education Research

European Journal of Physics

Harvard Educational Review

Higher Education

International Journal of Electrical Engineering Education

International Journal of engineering Education

International Journal of Science Education

International Journal of Sustainability in Higher Education

International Journal of Technology and Design Education

Journal for Research in Mathematics Education

Journal of Biological Education

Journal of Chemical Education

Journal of Economic Education

Journal of Education Policy

Journal of Educational and Behavioral Statistics

Journal of Engineering Education

Journal of Environmental Education

Journal of Geography in Higher Education

Journal of Higher Education

Journal of Materials Education

Journal of Planning Education and Research

Journal of Professional Issues in Engineering Education and Practice

Journal of Research in Science Teaching

Journal of Research on Educational Effectiveness

Journal of Studies in International Education

Journal of Teacher Education

Mathematical Thinking and Learning

Minerva

Physical Review Special Topics-Physics Education Research

Research in Higher Education

Research in Science Education

Review of Educational Research

Review of Higher Education

Revista Latinoamericana de Investigacion en Matematica Educativa

Science and Education

Science Education

Sociology of Education

Studies in Higher Education

Studies in Science Education

Teaching and Teacher Education

Teaching of Psychology

Teaching Sociology

Thinking Skills and Creativity

Vocations and Learning

**Journals assigned to Border Fields**

Applied Measurement in Education

Australasian Journal of Educational Technology

British Journal of Educational Psychology

British Journal of Educational Technology

Cognition and Instruction

Computer Assisted Language Learning

Computers and Education

Contemporary Educational Psychology

Creativity Research Journal

Discourse Processes

Distance Education

Early education and Development

Educational and Psychological Measurement

Educational Psychologist

Educational Psychology

Educational Psychology Review

Educational Technology and Society

Educational Technology Research and Development

European Journal of Psychology of Education

Gifted Child Quarterly

High Ability Studies

IEEE Transactions on Education

IEEE Transactions on Learning Technologies

Infancia y Aprendizaje

Instructional Science

Interactive Learning Environments

International Journal of Bilingual Education and Bilingualism

International Journal of Computer-Supported Collaborative Learning

International Review of Research in Open and Distance Learning

Internet and Higher Education

Journal of Computer Assisted Learning

Journal of Computing in Higher Education

Journal of Creative Behavior

Journal of Diversity in Higher Education

Journal of Educational Computing Research

Journal of Educational Measurement

Journal of Educational Psychology

Journal of Experimental Education

Journal of Literacy Research

Journal of Psychoeducational Assessment

Journal of Research in Reading

Journal of Science Education and Technology

Journal of the Learning Sciences

Language Assessment Quarterly

Language Learning

Language Learning and Technology

Language Teaching

Language Teaching Research

Learning and Individual Differences

a. Learning and Instruction

b.Learning media and Technology

c.Metacognition and Learning

d.Mind brain and Education

e.Modern Language Journal

f.Psychologie in Erziehung und Unterricht

g.Psychology in the Schools

h.Psychology of Music

i.Reading and Writing

j.Reading Research Quarterly

k.Recall

l.Revista de Psicodidactica

m.Scientific Studies of Reading

n.Social Psychology of Education

o.System

p.Technology Pedagogy and Education

q.Tesol Quarterly

r.Voprosy Psikhologii

s.Zeitschrift fur Entwicklungspsychologie und Padagogische Psychologie

Zeitschrift fur Padagogische Psychologie
