# Supplementary material for: A measure of knowledge flow between specific fields: Implications of interdisciplinarity for impact and funding
Source: PLoS One. 2017 Oct 9;12(10):e0185583. doi: 10.1371/journal.pone.0185583 (PMC5633153; doi:10.1371/journal.pone.0185583)
Supplement: S2 File — (DOCX) [file pone.0185583.s002.docx]

**Appendix1. Number of Received Citations per Article**

Number of received citations per article by discipline and publication year (x>0%)

|  |  | CogSci | | ED | | Border | |
| --- | --- | --- | --- | --- | --- | --- | --- |
| Year | Statistics | Non-KMED | A-type | Non-KMED | A-type | Non-KMED | A-type |
| 1994 | Obs. | 1,033 | 674 | 486 | 362 | 169 | 596 |
|  | Average | 38.4 | 90.0 | 7.5 | 28.7 | 4.7 | 27.5 |
|  | Median | 22 | 44.5 | 3 | 15 | 1 | 11 |
| 1999 | Obs. | 1,356 | 755 | 716 | 468 | 113 | 690 |
|  | Average | 35.2 | 71.8 | 10.3 | 24.1 | 5.0 | 29.1 |
|  | Median | 22 | 43 | 6 | 14 | 3 | 13 |
| 2004 | Obs. | 1,619 | 831 | 789 | 651 | 166 | 797 |
|  | Average | 32.7 | 52.6 | 8.1 | 21.5 | 6.3 | 28.2 |
|  | Median | 21 | 33 | 5 | 12 | 2.5 | 14 |
| 2009 | Obs. | 2,656 | 1,108 | 1,094 | 1,135 | 330 | 1,795 |
|  | Average | 15.7 | 22.0 | 4.8 | 10.7 | 4.9 | 11.7 |
|  | Median | 11 | 15 | 3 | 7 | 3 | 7 |
| 2014 | Obs. | 2,895 | 1,458 | 1,384 | 1,686 | 324 | 2,326 |
|  | Average | 0.8 | 1.0 | 0.2 | 0.3 | 0.3 | 0.4 |
|  | Median | 0 | 0 | 0 | 0 | 0 | 0 |

Number of received citations per article by discipline and publication year (x=5%)

|  |  | CogSci | | ED | | Border | |
| --- | --- | --- | --- | --- | --- | --- | --- |
| Year | Statistics | Non-KMED | A-type | Non-KMED | A-type | Non-KMED | A-type |
| 1994 | Obs. | 1,496 | 348 | 588 | 253 | 225 | 523 |
|  | Average | 61.7 | 87.8 | 11.3 | 29.6 | 7.3 | 29.1 |
|  | Median | 30 | 45 | 5 | 14 | 1 | 11 |
| 1999 | Obs. | 1,946 | 326 | 864 | 315 | 187 | 590 |
|  | Average | 52.7 | 71.0 | 13.0 | 24.3 | 11.6 | 30.2 |
|  | Median | 29 | 40 | 7 | 15 | 4 | 14 |
| 2004 | Obs. | 2,237 | 337 | 985 | 436 | 255 | 666 |
|  | Average | 41.9 | 52.9 | 12.3 | 19.8 | 11.6 | 29.0 |
|  | Median | 25 | 33 | 5 | 11 | 4 | 15 |
| 2009 | Obs. | 3,406 | 356 | 1,490 | 695 | 582 | 1,443 |
|  | Average | 18.2 | 19.3 | 6.4 | 10.9 | 7.4 | 12.0 |
|  | Median | 12 | 13 | 4 | 7 | 4 | 7 |
| 2014 | Obs. | 3,885 | 463 | 2,082 | 984 | 781 | 1,859 |
|  | Average | 0.8 | 0.9 | 0.3 | 0.3 | 0.3 | 0.4 |
|  | Median | 0 | 0 | 0 | 0 | 0 | 0 |

Number of received citations per article by discipline and publication year (x=10%)

|  |  | CogSci | | ED | | Border | |
| --- | --- | --- | --- | --- | --- | --- | --- |
| Year | Statistics | Non-KMED | A-type | Non-KMED | A-type | Non-KMED | A-type |
| 1994 | Obs. | 1,740 | 146 | 699 | 152 | 370 | 362 |
|  | Average | 62 | 100 | 14 | 34 | 13 | 31 |
|  | Median | 31 | 51 | 7 | 13 | 1 | 12 |
| 1999 | Obs. | 2,209 | 131 | 1,010 | 184 | 309 | 406 |
|  | Average | 54 | 73 | 15 | 24 | 15 | 33 |
|  | Median | 30 | 39 | 7 | 16 | 6 | 16 |
| 2004 | Obs. | 2,492 | 125 | 1,178 | 264 | 413 | 479 |
|  | Average | 43 | 59 | 14 | 19 | 17 | 29 |
|  | Median | 26 | 35 | 7 | 10 | 6 | 15 |
| 2009 | Obs. | 3,720 | 124 | 1,772 | 398 | 964 | 955 |
|  | Average | 19 | 20 | 7 | 12 | 9 | 12 |
|  | Median | 12 | 14 | 4 | 7 | 5 | 7 |
| 2014 | Obs. | 4,170 | 165 | 2,501 | 554 | 1,338 | 1,283 |
|  | Average | 1 | 1 | 0 | 0 | 0 | 0 |
|  | Median | 0 | 0 | 0 | 0 | 0 | 0 |

Number of received citations per article by discipline and publication year (x=20%)

|  |  | CogSci | | ED | | Border | |
| --- | --- | --- | --- | --- | --- | --- | --- |
| Year | Statistics | Non-KMED | A-type | Non-KMED | A-type | Non-KMED | A-type |
| 1994 | Obs. | 1,902 | 33 | 820 | 57 | 612 | 137 |
|  | Average | 65.4 | 118.8 | 16.6 | 24.5 | 19.1 | 34.8 |
|  | Median | 33 | 43 | 8 | 11 | 7 | 14 |
| 1999 | Obs. | 2,371 | 23 | 1,158 | 62 | 586 | 146 |
|  | Average | 55.1 | 63.0 | 16.5 | 17.6 | 21.9 | 41.4 |
|  | Median | 30 | 39 | 8 | 7 | 9 | 19 |
| 2004 | Obs. | 2,649 | 31 | 1,375 | 90 | 760 | 149 |
|  | Average | 43.0 | 63.8 | 15.0 | 16.4 | 24.3 | 26.3 |
|  | Median | 26 | 31 | 7 | 8 | 11 | 14 |
| 2009 | Obs. | 3,908 | 17 | 2,062 | 109 | 1,616 | 285 |
|  | Average | 18.8 | 24.8 | 7.7 | 12.7 | 10.9 | 11.6 |
|  | Median | 12.5 | 13 | 5 | 8 | 6 | 7 |
| 2014 | Obs. | 4,290 | 41 | 2,888 | 158 | 2,214 | 387 |
|  | Average | 0.8 | 1.1 | 0.3 | 0.3 | 0.3 | 0.5 |
|  | Median | 0 | 0 | 0 | 0 | 0 | 0 |

**Appendix2. Share of Articles with Funding Acknowledgement**

| x>0% | non-KMED | KMED | A-type | B-type | D-type |
| --- | --- | --- | --- | --- | --- |
| Border | 11.8% | 6.3% | 6.3% | 5.6% | 6.0% |
| CogSci | 48.0% | 26.6% | 26.3% | 26.2% | 20.5% |
| ED | 22.4% | 10.8% | 11.0% | 7.5% | 2.3% |
|  |  |  |  |  |  |
| x=5% | non-KMED | KMED | A-type | B-type | D-type |
| Border | 10.6% | 5.7% | 5.5% | 5.4% | 4.9% |
| CogSci | 44.6% | 15.1% | 13.6% | 15.4% | 15.6% |
| ED | 19.4% | 9.8% | 10.2% | 6.7% | 2.1% |
|  |  |  |  |  |  |
| x=20% | non-KMED | KMED | A-type | B-type | D-type |
| Border | 8.2% | 2.8% | 3.0% | 2.4% | 1.0% |
| CogSci | 41.4% | 12.6% | 13.8% | 12.9% | 10.9% |
| ED | 16.9% | 7.8% | 10.5% | 4.3% | 1.7% |
